# An atlas on biomarkers for Alzheimer's disease

**DOI:** 10.3389/fnagi.2013.00054

**Published:** 2013-11-08

**Authors:** Marta Martínez-Rivera

**Affiliations:** Hospital Álvarez-BuyllaMieres, Spain

**Keywords:** Alzheimer, biomarker, dementia, mild cognitive impairment, atlas, differential diagnosis, book review, treatment

The search of methods to ease the diagnosis of Alzheimer's disease (AD) as early as possible is in the center of attention. Such methods (biomarkers) are a range of blood or CSF tests on one hand, and several types of neuroimaging scans on the other. Biomarkers are going to be included not only in the neuroscience research area but also in the diagnosis process with a practical application in the next years. The potential for these biomarkers to serve diagnostic purposes of AD has been highlighted in the proposed diagnostic criteria for preclinical AD from an NIH/NIA working group (Sperling et al., 2011). In these criteria, biomarkers are defined in terms of whether they reflect Aβ deposition, tau deposition, or signs of neuronal injury. Markers of Aβ deposition include both positron-emission tomography (PET) evidence of Aβ deposition and cerebrospinal fluid (CSF) measures of lower Aβ42 levels, using a variety of specific ligands. Markers of tau accumulation include CSF measures of increased total tau or phosphorylated-tau (p-tau). Together with low CSF Aβ42, elevated CSF tau provides a high likelihood of progression to AD in patients with MCI (mild cognitive impairment). A third group of biomarkers reflect biochemical changes related to processes such as cell death, synaptic damage, oxidative stress, or inflammation that may be part of the cascade of events that mediate damage, or the response to damage, in AD. This is a field, as many others in science, that suffers a quick evolution every year; and the moment of apply biomarkers in clinical practice is coming. It is necessary to make the clinical community aware of these scientific advances in a clear and concise manner, as well as counting with references that can guide our clinical practice with a consensus point of view.

Many of the images coming both from laboratory and neuroimaging studies are very illustrative. These images, accompanied by a short description, can perfectly explain the main results and usefulness of each biomarker. And this is just what the Atlas created by Dr Manuel Menéndez does (Menéndez González, 2011). The objective of this book is to summarize the most important studies made in this field. Few publications have systematically compiled results on this topic and none as an atlas. The book starts clarifying concepts as “biomarker”, “mild cognitive impairment” and other preclinical conditions, and then focus on classification and description of all biomarkers for AD. A collection of imagines selected from outstanding research studies are provided for both laboratory and neuroimaging biomarkers. Finally the author finishes with a chapter on the rational use of biomarkers for AD in the clinical setting.

Readers will be interested in this publication because it allows reviewing the current status of research at the time that visualizing outstanding results easily. The possibility of coming across an optimal and well done review of biomarkers for AD is crucial not only for expertees, but also for the large public seeking to make a first approach to the world of biomarkers for AD. Such an easy to use manual, with the purpose to make the broad and often confusing biomarkers discussion surrounding AD accessible to a wider audience, notably in the clinic, is invaluable. The contents seem adequate, and Dr. Menéndez has a well-established track record in this field that ensures the quality of the final product, and therefore I am certain the scientific contents are solid. Overall it is a solid and timely proposal, at a competitive price range, which I am sure will make an impact where it matters the most, in the clinic with patients.
